# Precision Estimation of Rice Nitrogen Fertilizer Topdressing According to the Nitrogen Nutrition Index Using UAV Multi-Spectral Remote Sensing: A Case Study in Southwest China

**DOI:** 10.3390/plants14081195

**Published:** 2025-04-11

**Authors:** Lijuan Wang, Qihan Ling, Zhan Liu, Mingzhu Dai, Yu Zhou, Xiaojun Shi, Jie Wang

**Affiliations:** 1 College of Resources and Environment, Southwest University, Chongqing 400716, China; w892694385@163.com (L.W.);; 2 National Monitoring Station of Soil Fertility and Fertilizer Efficiency on Purple Soils, Chongqing 400716, China; 3 Menghai Manxiang Yuntian Agriculture Development Co., Ltd., Menghai 666205, China; 4 Chongqing Field Scientific Observatory of Soil Quality and Ecological Environment, Chongqing 400716, China

**Keywords:** UAV multi-spectral remote sensing, nitrogen nutrition index, precise nitrogen topdressing, random forest

## Abstract

The precision estimation of N fertilizer application according to the nitrogen nutrition index (NNI) using unmanned aerial vehicle (UAV) multi-spectral measurements remains to be tested in different rice cultivars and planting areas. Therefore, two field experiments were conducted using varied N rates (0, 60, 120, 160, and 200 kg N ha^−1^) on two rice cultivars, Yunjing37 (YJ-37, *Oryza sativa subsp. Japonica Kato.*, the Institute of Food Crops at the Yunnan Academy of Agricultural Sciences, Kunming, China) and Jiyou6135 (JY-6135, *Oryza sativa subsp. indica Kato.*, Hunan Longping Gaoke Nongping seed industry Co., Ltd., Changsha, China), in southwest China. The rice canopy spectral images were measured by the UAV’s multi-spectral remote sensing at three growing stages. The NNI was calculated based on the critical N (Nc) dilution curve. A random forest model integrating multi-vegetation indices established the NNI inversion, facilitating precise N topdressing through a linear platform of NNI-Relative Yield and the remote sensing NNI-based N balance approaches. The Nc dilution curve calibrated with aboveground dry matter demonstrated the highest accuracy (R^2^ = 0.93, 0.97 for shoot components in cultivars YJ-37 and JY-6135), outperforming stem (R^2^ = 0.70, 0.76) and leaf (R^2^ = 0.80, 0.89) based models. The RF combined with six vegetation index combinations was found to be the best predictor of NNI at each growing period (YJ-37: R^2^ is 0.70–0.97, RMSE is 0.02~0.04; JY-6135: R^2^ is 0.71–0.92, RMSE is 0.04~0.05). The RF surpassed BPNN/PLSR by 6.14–10.10% in R^2^ and 13.71–33.65% in error reduction across the critical rice growth stages. The topdressing amounts of YJ-37 and JY-6135 were 111–124 kg ha^−1^ and 80–133 kg ha^−1^, with low errors of 2.50~8.73 kg ha^−1^ for YJ-37 and 2.52~5.53 kg ha^−1^ for JY-6135 in the jointing (JT) and heading (HD) stages. These results are promising for the precise topdressing of rice using a remote sensing NNI-based N balance method. The combination of UAV multi-spectral imaging with the NNI-nitrogen balance method was tested for the first time in southwest China, demonstrating its feasibility and offering a regional approach for precise rice topdressing.

## 1. Introduction

Rice serves as a primary food source for over 33% of the world’s population, and it is the leading food crop in China [1]. The southwestern region of China contributes 13.4% to the country’s total rice production [2]. Nitrogen (N) is a crucial input that significantly limits yield. The N applied to rice was 20.8% below the national average, leading to a corresponding decrease in yield in the southwestern region of China [3]. Improper N application not only prevents rice yields from reaching their maximum potential, but can also lead to the wastage of land resources and even present a potential threat to environmental health [4]. As a result, effective N management is crucial to achieve a balance between crop yield and sustainability.

Traditional fertilization practices are frequently guided by farmers’ practice or standardized recommendations, overlooking the dynamic process of N absorption in rice. This can lead to the incorrect use of N fertilizers. Precision agriculture is a management approach with the main goal of optimizing the management of agronomic inputs across both space and time to enhance N use efficiency (NUE) [5]. Currently, precise N application techniques involve the NNI method [6], leaf area index (LAI) [7], and N fertilizer optimization algorithm (NFOA) [8], along with simple redistribution functions [9] and mass balance principles [10]. The N amounts applied using the NNI and LAI methods were nearly identical, whereas the application rate for the NFOA was lower [11]. Higher LAI values showed greater sensitivity to changes in the crop’s N status, which negatively impacted sensor readings during the later stages of growth. The modified NFOA boosted yield by 29–48% without causing an N deficit [12]. However, this method has a limited capability to accurately quantify N use by crops, and it presents some deviations. Additionally, the simple redistribution function enables farmers to apply fertilizer using an uncomplicated linear algorithm [9]. Mass balance uses the protein content of the target yield as a standard to estimate the crop’s total N requirement. However, this approach may overlook the N supply in the soil [10]. Therefore, creating a straightforward and reliable model to assess a crop’s N nutrient status is essential for achieving success in using remote sensing for real-time monitoring.

To enable the swift and precise monitoring of a crop’s N nutritional status, the NNI was introduced in 1997 by [13]. The NNI serves as a valuable tool for effectively assessing the N nutritional status of crops. A value of NNI > 1 indicates a non-N-limiting condition, signifying an excess of N. Conversely, an NNI < 1 denotes an N-limiting condition, indicating insufficient N levels as the plants’ N concentration falls below the curve. The conventional method for determining N concentration lacks timeliness and is destructive to rice growth. By integrating the NNI method with remote sensing technology, the real-time N nutrition status of rice can be effectively monitored. This approach allows for a comprehensive understanding of the interactions between N nutrition, genotype, environment, and management practices [14]. Previous researchers have utilized vegetation indices to monitor crop N nutrition, finding a strong correlation with NNI [15]. Among them, the normalized differential vegetation index (NDVI) and soil regulates vegetation index (SAVI) were widely used to assess N nutrition in crops like maize [16], wheat [17], and rice [18] at the canopy level. The NDVI and ratio vegetation index (RVI) were used to analyze the N conservation and NNI of tomatoes at various growth stages [19]. Their findings revealed the ability of NDVI and RVI to precisely predict the N conservation and NNI of the plants at different growth stages. Furthermore, researchers have identified vegetation indices such as the normalized color difference index (PSNDc) and SAVI as having strong correlations to NNI and minimal multi-collinearity [15]. Considering the limitations of individual vegetation indices in band selection and spectral representation, combining multiple indices represents a novel approach to N model inversion. UAV images were used to monitor rice crop NNI and optimize the model R^2^ with a Random Forest Regressor (RF) from 0.88 to 0.96 [20].

Currently, the application of unmanned aerial vehicle (UAV) based multi-spectral remote sensing to monitor rice nitrogen (N) status has been widely studied, but further precision estimation of N fertilizer application by NNI using UAV multi-spectral imaging remains to be tested in different cultivars and planting areas. The objectives of this study are: (1) Calculate the Nc dilution curve and create the linear platform of NNI-Relative Yield (Ry); (2) Develop an inversion model for NNI using sensitive vegetation indices and machine leaning; (3) Establish a remote sensing NNI-based N balance method for in-season rice N diagnosis and precise N fertilizer topdressing.

## 2. Materials and Methods

This study was conducted at two sites, Mengzhe Town in Menghai County, Xishuangbanna Dai Autonomous Prefecture, Yunnan Province (abbreviated as MZF, 100°13′ E, 21°57′ N, 1265.08 m above sea level) and the National Monitoring Station of Soil Fertility and Fertilizer Efficiency on Purple Soils (abbreviated as BPS, 106°26′ E, 30°26′ N, altitude 317 m) situated in southwest China [21]. In this study, MZF belongs to a subtropical plateau monsoon climate. The region receives a 24.01 °C average temperature and 1526.2 mm of rainfall annually. The BPS belongs to the subtropical humid monsoon climate. The region receives an 18.5 °C average temperature and 1105.1 mm of rainfall annually. Table 1 shows the detailed soil physical and chemical properties and the seeding method used at the two experimental sites.

Five varied N fertilizer rates ranging from 0 to 200 kg·N·ha^−1^ were imposed as N treatments (Table 2), expressed by N0, N1, N2, N3 and N4, respectively. The size of every plot was 50 m^2^. An inter-row spacing of 30 cm was used at both sites. The planting density was 25 × 10^4^ plants·ha^−1^ in all experiments, two seedlings per hole. The base fertilizer of the two varieties accounted for 40% of the total N. For YJ-37, N was applied at 40% and 20% of the total N during the jointing (JT) and heading (HD) stages, respectively. For JY-6135, N application at the tillering (TR) and JT stages represented 40% and 20% of the total N, correspondingly. In each experiment, every plot received 120 kg P_2_O_5_ ha^−1^ and 105 kg K_2_O ha^−1^ before transplantation.

A DJI Phantom 4 pro (DJI Company, Shenzhen, China) multi-spectral UAV was used for this experiment; its parameters are shown in Table 3. The acquisition times of multi-spectral images of the rice canopy were the TR, JT, HD, and filling stage (FL), respectively. Images were collected under stable low wind, cloudless, and sunny-sky conditions from 10:00 to 14:00. Reflectance calibration was collected with standard gray cloth (2 m × 2 m) before each flight. The UAV flew vertically over the ground, setting a flight height of 100 m, a course overlap rate of 80%, and a side overlap rate of 70%. The camera is set to take pictures at each waypoint automatically, and the images are saved in TIFF format. There were five wavebands: 450 nm (Blue, B), 560 nm (Green, G), 650 nm (Red, R), 730 nm (Red edge, RE), and 840 nm (Near-infrared, NIR) used to construct the vegetation index. We rebuilt the image using DJI TerraV3.6.0 software. We then incorporated the radiation correction parameters supplied by the standard gray cloth. The next step was to generate a multi-spectral orthophoto with reflectance values across five bands. The rice images at TR were processed with masking techniques to eliminate soil background values and retrieve the spectral data of the rice canopy.

Destructive sampling was conducted at each key stage of rice growth, including the TR, JT, HD and FL periods. Six representative plants were randomly sampled. The plant samples were divided into leaf (green leaf blade), stem (culm plus sheath) and panicle. All the samples were oven-dried for 30 min at 105 °C and then at 70 °C to a constant weight to attain the plant dry matter (DM, t ha^−1^). All samples were ground to 1 mm using a Wiley mill to determine the plant’s N concentration (PNC, %) using the Kjeldahl–N method.

The plants’ N accumulation (PNA) at each growth stage (kg·ha^−1^) was calculated as follows:

(1) $$\mathrm{PNA}=L_{W}L_{N}+S_{W}S_{N}+P_{W}P_{N}$$

where L_W_, S_W_ and P_W_ are the dry weights of leaves, stem, and panicle (kg·ha^−1^) while L_N_, S_N_ and P_N_ are the N concentrations of leaves, stem, and panicle (%), respectively.

According to the method proposed by Justes et al. [22], constructing the Nc dilution curve mainly includes the following steps: (1) The shoot DM and N concentration data for the different N treatments were compared by performing an analysis of variance (ANOVA) using SPSS Software 27.0 (IBM, Armonk, NY, USA) at the 5% probability level. These data points were used to either construct or validate the N dilution curve. (2) Data for each sampling date were divided into two groups: (i) The N-limited group, where increasing the N supply caused a significant change in both shoot DM and N concentration; (ii) The non-N-limited group, where increasing the N supply did not increase shoot DM but did increase N concentration. (3) The data for the N-limited group were fitted using a simple linear regression (an oblique line), whereas the data for the non-N-limited group were used to calculate the maximum shoot DM from the mean of the observed data (represented by a vertical line). (4) The theoretical N point was defined as the ordinate value of the intersection between the oblique and vertical lines. The allometric equation for the line fitted to the critical points was derived using a previously published description of the N concept [23].

The Nc dilution curve model based on leaf dry matter constructed by the above method is as follows:

Nc = a × LDM^−b^ (2)

Nc is the critical N concentration of rice (%) and LDM is the dry matter weight of leaves (t·ha^−1^). The term a is the critical N concentration (%) of leaf dry matter of 1 t·ha^−1^; b is the exponential slope of the N dilution curve.

According to Lemaire et al. [13], the model of NNI expression is as follows:

(3) $$\mathrm{NNI}=\frac{Na}{Nc}$$

where Na represents the measured N concentration of rice leaves (%) and Nc stands for critical leaf N concentration (%).

The correlations between NNI and the vegetation indexes of other crops were statistically summarized by previous studies on rice [24,25], wheat (*Triticum aestivum* L.) [15] and maize [16]. Nine types of NNI-sensitive vegetation index were selected (Table 4) and the multi-spectral reflectance data of three growth stages of two rice varieties were extracted. Using gray correlation analysis, a vegetation index that exhibited a strong degree of correlation was chosen as the input variable. A total of 80 sets of data were utilized, of which 70% of the data were randomly chosen as the training set to construct the rice NNI inversion model, and the remaining 30% of the data was used as the testing set in this paper. The NNI inversion model was constructed and verified by a random forest regression [20]. For the RF model construction, after parameter optimization and multiple training iterations, the number of decision trees was set to 100 for the dry matter (DM) models [26]. Additionally, a default value of 2 was applied to the maximum depth, minimum samples split and minimum samples leaf.

Gray correlation analysis (GCA) primarily functions as a technique for identifying the most significant factors by employing various approaches to clarify the key relationships among different variables and then grasp the key part of the contradiction [33]. In this study, the aboveground NNI of rice was utilized as the reference series, while the above nine vegetation indices were selected as the comparative series for conducting the gray correlation analysis. The specific procedures are outlined below:

(1) A non-dimensional approach was applied to the aboveground N nutrition index of rice.

(2) Calculation of the correlation coefficient was performed. By using the aboveground NNI of rice alongside the nine common vegetation indices, the gray correlation coefficients for both were computed according to the formula provided below.

(4) $$\gamma_{oi}(k)=\frac{∆min+\rho ∆max}{{∆}_{oi}\left(k\right)+\rho ∆max}$$

where, $\gamma_{oi}(k)$ is the correlation coefficient between the reference sequence and the comparison sequence *k*; Δmin and Δmax are the minimum absolute difference and the maximum absolute difference, respectively. ρ is the resolution coefficient, the value range is [0, 1], and ρ = 0.5 in this paper.

(3) Calculation of gray correlation degree (GCD)

(5) $$\gamma_{i}=\frac{1}{n}\sum_{i=1}^{n}\gamma_{oi}(k)$$

(4) Gray correlation degree ranking

According to the magnitude of GCD $\gamma_{i}$, the nine vegetation indices were ranked exponentially.

Root Mean Square Error (RMSE):

(6) $$\mathrm{RMSE}=\sqrt{\frac{1}{N}\sum_{i=1}^{n}{\left(Yi-f\left(xi\right)\right)}^{2}}$$

where Yi represents the measured value, f(xi) represents the inverse value, and n is the number of samples. The smaller the value of RMSE, the higher the accuracy of the rice physiological parameter inversion model.

In the NNI-based N balance method, the first step is to examine the relationship between relative yield (Ry) and the N nutrition index (NNI). The linear platform relationship between NNI and relative yield illustrates the threshold effect of the crop’s N use, providing a quantitative tool for the scientific management of N fertilizers.

RY = a × NNI − b (NNI < NNI_max_) (7)

RY = c (NNI > NNI_max_) (8)

where NNI_max_ is the NNI value that does not change significantly under the N gradient and parameters a, b and c are constants.

The NNI predicted RY during the critical growth period.

GY = Y max × RY(9)

The relationship between relative yield (Ry) and historical maximum yield (Y max) is used to predict the yield.

(10) $$\mathrm{GNA}=\mathrm{APGN}\;\times \;\frac{GY}{100}$$

Among them, APGN is the N absorption per 100 kg grain at a high yield level.

(11) $$\mathrm{Nr}=\frac{GNA-NS}{NUE}-\mathrm{Nuse}$$

where Nr is the amount of fertilizer applied in the field (kg·ha^−1^), NUE is the N utilization rate with a fixed value of 0.426 [32], Ns is the N uptake of plants treated in the soil control area (N0), and Nuse is the amount of N applied.

Equations (7)–(11) and historical data were used to quantify each formula and establish an N fertilizer control method based on NNI [32].

## 3. Results

### 3.1. Model of Critical N Dilution Curves and the Relative Yield

The increase of the aboveground N accumulation and yield followed a continuous increasing trend with increasing N application rates for both varieties (Table 5). There was no significant difference in N uptake of 100 kg grains. The N accumulation of YJ-37 and JY-6135 were 128.9 and 118.56 kg·ha^−1^ under N0 treatment, respectively. The N uptake required for 100 kg of grain to achieve the highest yield was 1.97 kg and 2.13 kg, respectively.

N rate represents the N application rate. TAN represents the aboveground N accumulation. APGN represents the N absorption of 100 kg of grain. The letters indicate the significant differences among different treatments (*p* < 0.05). Significant results of one-way ANOVA are shown.

**Figure 1:**
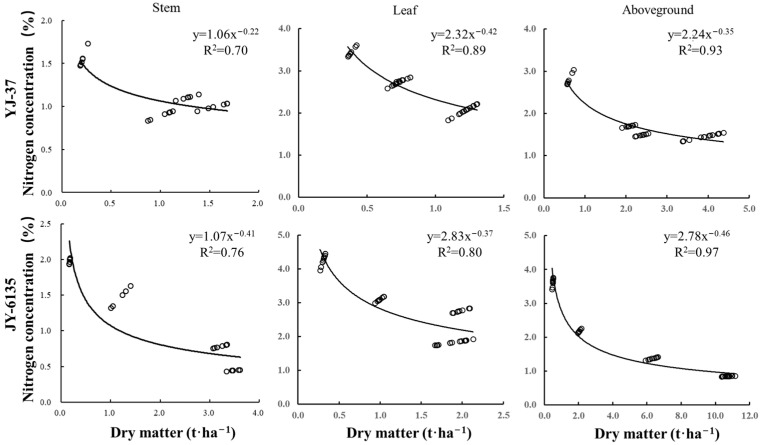
Critical nitrogen dilution curves of different rice cultivars based on stem, leaf and plant N concentration.

The Nc dilution curve was decreased with the increase in different parts’ dry matter of the two rice cultivars (Figure 1, Table A1 and Table A2), which satisfied a power function relationship. Parameters a and b of the Nc curve based on stem dry matter were lower than those of leaves and plants, mainly because the N uptake of stems was lower than those of the leaves and ground parts and the N concentration decreased slowly with the increase of dry matter. Parameter a was related to the initial N uptake capacity and soil N supply at the early stage of crop growth. Parameter b was related to the rate at which N concentration decreased with the increase of dry matter. The parameters a of the critical N concentration curves of YJ-37 based on stem, leaf and aboveground dry matter were 1.06, 2.32 and 2.24, and the parameters b were 0.22, 0.42 and 0.35, respectively. The parameters a of the Nc curves based on stem, leaf and aboveground dry matter of JY-6135 were 1.07, 2.83 and 2.78, and the parameters b were 0.41, 0.37 and 0.46, respectively. The accuracy of the Nc dilution curve based on aboveground dry matter R^2^ (0.93 and 0.97, respectively) was higher than that of the stem and leaf at 0.23, 0.21 and 0.04, 0.17, respectively.

**Figure 2:**
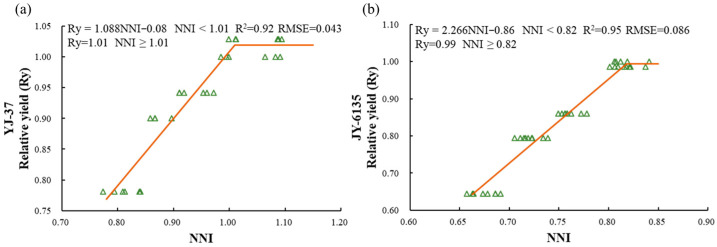
Relationship between relative yield (Ry) and nitrogen nutrition index(NNI) of YJ-37 and JY-6135 at different crop growth stages, jointing stage (**a**), tillering stage (**b**). The triangles in the figure are sample values.

**Figure A1:**
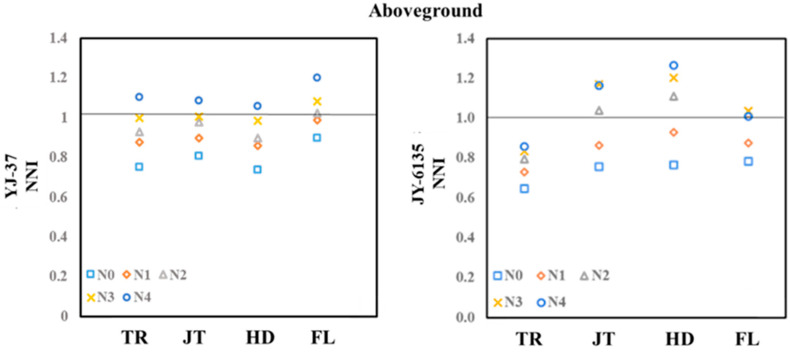
Aboveground nitrogen nutrient indices of YJ-37 and JY-6135 under different fertilization treatments.

The relationship between the NNI and Ry of the two rice varieties showed a linear platform relationship (Figure 2, Figure A1). When the NNI is below the critical value, yield can be significantly improved by increasing N fertilizer application. However, once the NNI reaches or exceeds the critical value, further fertilization will not lead to higher yields. For YJ-37, when the NNI < 1.01, the relative yield equals 1.088 × NNI − 0.08, and when the NNI ≥ 1.01, the relative yield is 1.01. For JY-6135, when the NNI < 0.82, the relative yield is 2.266 × NNI − 0.86, and when the NNI ≥ 0.82, the relative yield is 0.99.

### 3.2. Inversion Model of N Nutrient Index by Vegetation Indexes

**Figure 3:**
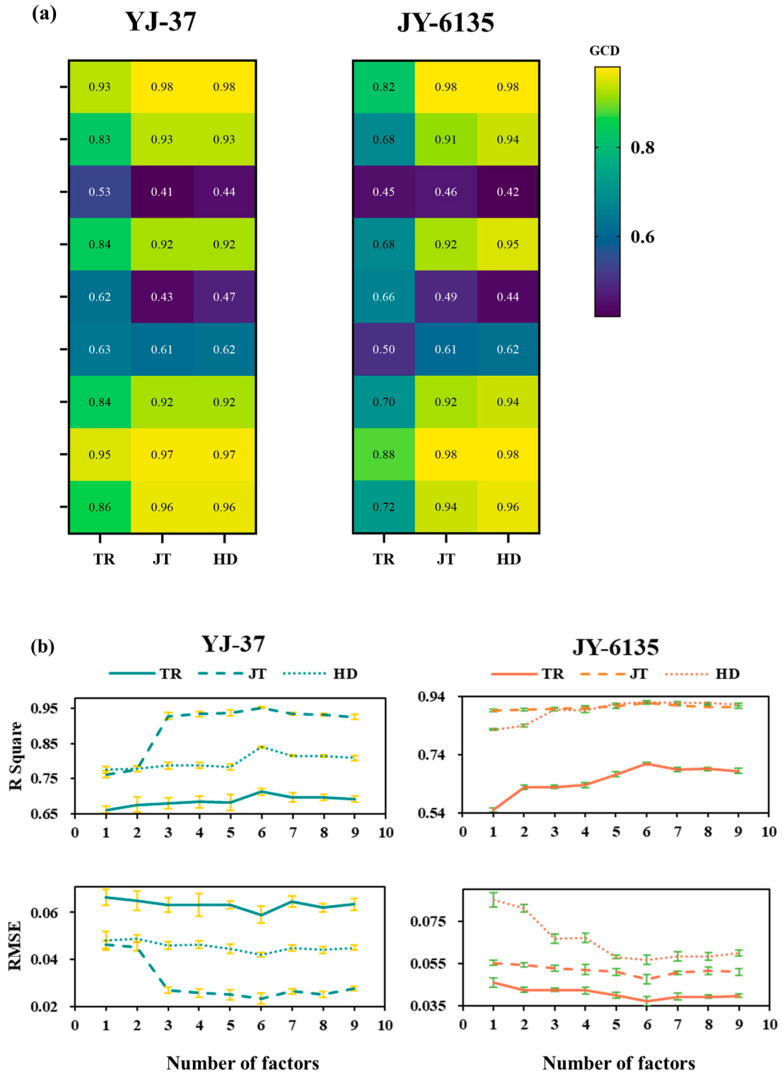
Gray correlation coefficient between the vegetation index and NNI of rice plants and its combined NNI inversion model accuracy and RMSE in each period. (**a**) Gray correlation coefficient between the vegetation index and NNI of rice plants. A lower value and darker color indicate a weaker gray correlation degree (GCD). (**b**) The R-squared and RMSE values of the Inverse NNI for various combinations of vegetation indices.

The vegetation indices constructed from the multi-spectral data were subject to GCA with the rice NNI for all the growth stages. As shown in Figure 3a, the gray correlation degree between the normalized vegetation index and the NNIs of YJ-37 and JY-6135 in each growth stage is higher than that of the ratio vegetation index, in which the correlation degree of NDVI, NNVI, NRI, NDRE, PSNDc and SAVI in each stage is higher than 0.7, and the other gray correlation coefficients are lower (between 0.41 and 0.66). Based on the findings of the gray correlation coefficients, various combinations of vegetation indices were modeled, and it was found that the NNI accuracy and RMSE obtained from the inversion of six vegetation indices as combinations reached the optimal level (Figure 3b). Consequently, the NNI inversion model of six vegetation index combinations was carried out by using RF in this study.

**Figure 4:**
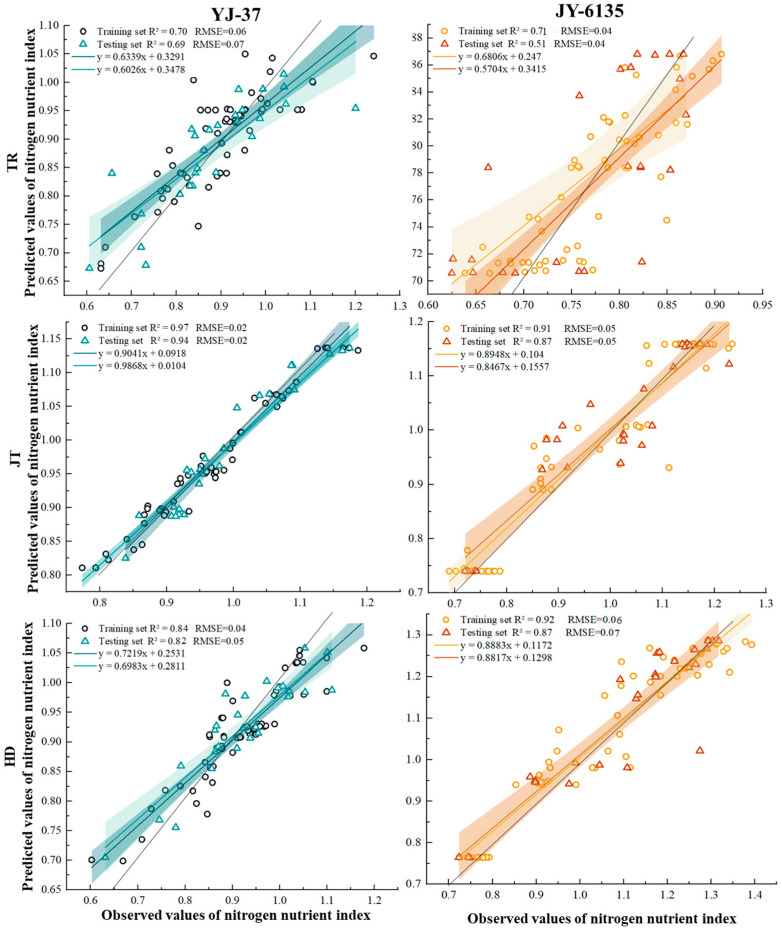
Relationship between predicted and measured NNI values of YJ-37 and JY-6135 at different growth stages.

The predicted values obtained by the RF of the two rice ecotypes were used as input variables, and then the final prediction results were obtained (Figure 4). The shaded area is the confidence interval (95%) of the inversion results. The results showed that the sample points of the two rice varieties were mainly within the 95% confidence interval, and the k coefficient was close to 1 at the JT and HD, which means the prediction was considered accurate. The YJ-37 training set exhibited accuracy within the range from 0.634 to 0.904, with R^2^ varying from 0.703 to 0.968 and RMSE from 0.024 to 0.063. In the testing set, the accuracy spanned from 0.603 to 0.987, R^2^ from 0.694 to 0.940, and RMSE from 0.023 to 0.072. For the JY-6135 training set, accuracy ranged from 0.681 to 0.913, R^2^ from 0.705 to 0.922, and RMSE from 0.041 to 0.058. In the testing set, accuracy varied between 0.570 and 0.873, R^2^ between 0.512 and 0.873, and RMSE between 0.044 and 0.069. In summary, the models of YJ-37 and JY-6135 have good accuracy and reliability in each period, so the two varieties have good predictive ability for the NNI in the three periods.

### 3.3. Field Fertilizer Application of Rice Based on NNI and Ry

**Figure 5:**
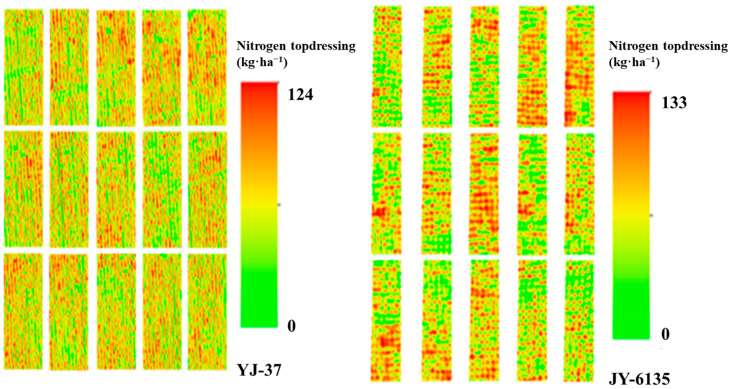
N topdressing at different test sites. The more intense the red color, the higher the value, indicating a greater nitrogen fertilizer application.

The proposed N control method in this paper is grounded on the linear platform connection between NNI and Ry, achieving the rational adjustment of target yield in accordance with the crop growth trend in the field. Subsequently, it provides appropriate topdressing recommendations based on the NNI-based N balance method. With the increase of the supplemental N amount followed a continuous increasing trend with increasing and then decreasing N application rates (Table 6). The complete N application for YJ-37 to achieve high yields was 191 kg·ha^−1^, and the total N application rate for JY-6135 to reach high yields was 197 kg·ha^−1^. The topdressing amounts of YJ-37 and JY-6135 were 111–124 kg·ha^−1^ and 80–133 kg·ha^−1^, respectively, and except for the TR of JY-6135, other topdressing errors were controlled within 2.5–8.73 kg·ha^−1^ and 2.52–5.53 kg·ha^−1^, respectively. These rates closely aligned with the suitable N rate illustrated in the Nc dilution curve, suggesting the effectiveness of the N control model developed in this research and offering substantial technical assistance for N fertilizer management in rice cultivation in southwestern China. According to the N operation ratio of this experiment, the recommended JT and HD fertilizer topdressing amounts of YJ-37 and the recommended TR and JT fertilizer topdressing amounts of JY-6135 are shown in Table 6. The inversion diagram of N topdressing for the two rice varieties is shown in Figure 5.

## 4. Discussion

The agronomic advantages of N application in enhancing rice production are well-established, with the Nc concentration dilution curve being a key tool for diagnosing crop N nutrition that is widely applied across various countries. Early research suggested that the N concentration dilution curve could be developed using the dry matter of various crop components [22]. In this study, the coefficient of determination for the Nc curve derived from aboveground biomass (YJ-37: 0.93, JY-6135: 0.97) outperformed that of the Nc curve for leaves and stems (Figure 1). This difference primarily arises because N stored in leaves and stems is gradually redirected to the ears during the late growth stages of rice, fulfilling grain filling requirements, resulting in significant N dilution in leaves and stems [34]. The japonica curve applies to aboveground DM values within the range of 0.34 to 12.47 t hm^−2^. The indica curve applies to aboveground DM values within the range of 0.49 to 10.70 t hm^−2^. Earlier research has demonstrated that rice ecotypes exhibit varying levels of adaptability to different climate types [34]. Indica rice is highly suited to tropical climates, though it also flourishes in subtropical conditions. On the other hand, japonica rice is specifically only adapted to subtropical areas and is easily distinguished by its strong reaction to N fertilizer [35]. This study presents newly established Nc curves for rice of various ecotypes in subtropical regions. The curve coefficients of a and b were 2.24 and 0.35 for japonica and 2.78 and 0.46 for indica rice, respectively. These newly generated curves were compared to those of tropical indica rice (5.18, 0.52) reported by Sheehy et al. [36] and subtropical japonica rice (3.53, 0.28) and indica rice (4.07, 0.28) from Ata-Ul-Karim et al. [34]. Significant differences were found among them. This disparity may be attributed to the influence of climate, as japonica rice is inherently suited to subtropical conditions, while indica rice also adapts well to such climates. Furthermore, the “a” value in this study is lower than that of the curve representing a subtropical environment. This could be attributed to the low N input from field management practices, which reduces the N absorption of rice. Additionally, the correlation between the initial N uptake capacity of crops and the N availability in the soil during the early growth stages may also explain this difference [37]. A previous study examined the combined impact of various factors and explored the uncertainty associated with rice parameters [38]. The study revealed that parameter a remained relatively stable across regions, years and management practices within the same climate type. Parameter b, in contrast, shows greater sensitivity to both genetic and environmental factors, with its coefficient of variation reaching 23.82% across different environmental conditions [38]. This study found no significant difference in parameter a between japonica and indica rice, although a small difference was observed in parameter b. This also suggests that the b value fluctuated considerably depending on the environmental conditions. An experimental study on N fertilization in wheat demonstrated that the interaction between genotype, environment and management had a slightly greater effect than parameter a. The planting environment exhibited a stronger response in terms of N absorption by wheat biomass, further corroborating the role of the planting environment on parameter b [39].

Although higher plant N levels are typically linked to increased yields, applying too much N fertilizer does not always lead to a higher crop yield. Numerous attempts have been made to estimate in-season RY based on the NNI of spring wheat, corn, sunflower, barley, and rice [40,41,42,43,44,45]. During the early stages of rice growth and development, the relationship between NNI and Ry can provide real-time, high-precision estimates of rice yield. In this study, linear relationships between NNI and Ry were developed for both japonica and indica rice during the JT and TR stages, respectively. The R^2^ values exceeded 0.92, while the RMSE ranged from 0.04 to 0.09. Additionally, the NNI levels observed on the platform fell within the optimal N nutrient range of 0.95 to 1.05, aligning with the findings of Xia et al. [46]. A widely used approach for predicting rice yield involves quantifying the connection between relative yield and NNI, accumulated N deficit (AND), and the N requirement (NR) through the application of the Nc dilution curve. The relationship established in this study indicates that inadequate fertilization during the early growth stages can result in a certain degree of yield loss. To prevent any negative impacts on the yield of food crops, it is crucial to restore non-limiting N nutrition levels (NNI = 1) prior to flowering [31,47]. Insufficient fertilization during the critical fertilization period for japonica rice and indica rice could decrease yields by 6–22% and 14–33%, respectively, thereby influencing the required amount of topdressing. The relationship of Ry to AND [45] is significant. The yields of both japonica and indica rice declined by 50% to 60%, with R^2^ values ranging from 0.94 to 0.99 and RMSEs between 7% and 9% during the optimal growth period, identified as the HD. The relationship of Ry to NR is also evident [45]. The yields of japonica and indica rice were reduced by 40% to 50%, the R^2^ values fell between 0.94 and 0.96, and the RMSE ranged from 5% to 7%. When comparing the three yield estimation techniques, it is evident that the accuracy and RMSE values attained by these methods are quite similar, while the linear platform relationship of NNI-Ry has a lower yield reduction than the other two methods and the estimation of topdressing amount is slightly better than the other two methods. Furthermore, regarding the acquisition of indices, NNI can attain accurate estimations without loss through multi-spectral NNI inversion. Conversely, the NR for crops is influenced by their N status and NRE, and its acquisition often necessitates destructive sampling methods. Thus, the linear plateau relationship between NNI and Ry could serve as a faster and more accurate method of predicting rice yield.

By utilizing remote sensing technology, spectral and image data can be gathered to develop an NNI fitting curve that enables the monitoring of N nutritional status [48]. This approach not only offers a rapid and real-time solution but also eliminates the need for destructive sampling. In this study, we conducted gray correlation analysis using nine existing vegetation indices and selected a combination of six vegetation indices for NNI inversion. Among these, the indices RVI and SR were significantly influenced by the relief of the terrain in our experimental field. Particularly, RVI, a sensitive parameter for assessing green plant health, was notably impacted by the red wavelength reflections from the crops [49]. Based on the findings of this research, the fitting performance of the six combinations of vegetation indices modeled by RF surpasses that of other combinations, providing a more accurate representation of crop growth during each critical fertilization period. The models of this study’s R^2^ values (YJ-37: 0.70~0.97; JY-6135: 0.71~0.92) and RMSE values (YJ-37: 0.02~0.04; JY-6135: 0.04~0.05) demonstrate significant improvements over previous results [18,25] from single spectral index NNI inversion (R^2^ ranging from 0.41 to 0.62, RMSE ranging from 0.15 to 0.21; R^2^ ranged from 0.24 to 0.90, RMSE ranged from 0.09 to 0.18), which indicates that the N topdressing error associated with the multi-vegetation index remote sensing fitting for NNI prediction in this study was less than that observed in earlier research. Furthermore, the K-coefficients are nearly equal to 1, indicating a superior fit, particularly during the JT and HD. This finding aligns with the exceptional fitting performance of the NDVI-NNI model developed [50] during the JT, which may be because when the vegetative growth of rice reaches its maximum, the multi-spectral imager acquires pure rice plant elements. As a result, an NNI inversion that relies on the vegetation index offers distinct advantages for diagnosing N levels and managing fertilization.

A major challenge to current N recommendation approaches is the difficulty of correctly assessing the yield target prior to the season. At present, four N-regulation methods have been extensively reported: NNI, LAI and NFOA [6,7,8,51]. Compared with other methods, the N balance method based on NNI comprehensively considered the N demand, yield, N use efficiency, soil N supply and N applied per amount of rice, the method used by predecessors is the same [6,32]. The method, combined with the non-destructive monitoring capabilities of remote sensing, enables real-time and accurate N recommendations for rice during the current season. Based on the topdressing amounts determined using this method, the total N applications for JY-37 and JY-6135 were 191 and 197 kg·hm^−2^, respectively, showing no significant difference from the initial N application amounts. Moreover, when the N concentration in the base fertilizer is insufficient, increasing the topdressing amount later in the growth period can significantly enhance ear development and grain filling. This practice helps mitigate the effects of inadequate fertilization during the earlier stages on overall yield [31,47]. Research has demonstrated that the N application determined using the NNI and LAI methods is nearly identical, whereas the N application amount calculated by the NFOA method is significantly lower [11,12]. We modified NFAO to achieve a yield increase of 29–48% without any damage to the N fertilizer. However, this approach is weak as a method of assessing the N status of crops and quantifying their N uptake, which may lead to some deviations. Zhang et al. [32] subsequently standardized SPAD readings based on prior research [51,52] and implemented the SPAD-based NFOA approach, enhancing N utilization efficiency by 3–9%. This method effectively mitigates the challenges posed by crop variety, geographical location, annual fluctuations and growth stages. Nevertheless, this method necessitates excessive N treatments to effectively normalize the N regulation reflected in SPAD values [53]. Additionally, the inversion NNI model indicates that the topdressing error for YJ-37 during the JT and HD stages is relatively small, with RMSE values maintained between 3.5 and 8.73 kg·hm^−2^. In conditions of inadequate TR N supply, the error for JY-6135 was more pronounced. The rice in its vegetative growth phase resulted in a less effective NNI inversion compared to the JT and HD periods, and during the initial growth phase of paddy fields, canopy reflectance is often affected by various background factors such as soil and water [54]. Furthermore, the reduction in yield associated with the established NNI-RY relationship during this timeframe may have contributed to a higher fertilizer application rate. For the HD period, RMSE was managed at 2.52 to 4.05 kg·ha^−1^. This outcome outperforms the results from Lu et al. [53], who predicted the Economic Optimal N Rate (EONR) using NNI and RF (R^2^ = 0.4, RMSE = 10.93 kg·ha^−1^). Thus, this study primarily advocates for employing the remote sensing NNI-based N balance method to estimate the topdressing amount between JT and HD. This approach ultimately enables non-destructive monitoring of N application levels for rice during the current growing season.

Expanding the method developed in this study to large, medium and small regions and areas is a promising and worthwhile task. Trials can be carried out at various agroecological sites to establish more consistent and dependable relationships between spectral indices and N indicators. Satellite remote sensing can be highly effective as a way of gathering crop data over extensive regions [55]. To calibrate satellite-based models for monitoring crop growth, single or multiple field measurement techniques are typically employed, though these methods can be time-intensive when it comes to non-destructive plant sampling. Handheld spectrometers are designed for precise analysis of small sample ranges, but they incur significant time and labor costs when applied to larger areas. Satellite and UAV technologies enable rapid and large-scale assessment of a crop’s N status during the jointing stages [56,57], which aids in providing recommendations for topdressing N application. Accurately determining the N requirements for smallholder farming remains a challenge. In the future, efforts are anticipated to focus on reducing the technical barriers for farmers by developing lightweight decision-making tools that support sustainable rice production.

## 5. Conclusions

This study established distinct Nc dilution curves for japonica (y = 2.24x^−0.35^, applicability range: 0.34–12.47 t ha^−1^) and indica (y = 2.78x^−0.46^, range: 0.49–10.70 t ha^−1^) rice cultivars in southwest China. The predicted R^2^ of NNI based on a multi-vegetation index inversion is 5.56–22.67% higher than that of the single vegetation index. The difference between the estimated total N application and the maximum yield was under 5%, validating the feasibility of the method. Using the results of NNI inversion alongside the nutrient balance method, optimal N application amounts (191 kg hm^−2^ for YJ-37 and 197 kg hm^−2^ for JY-6135) and topdressing amounts were determined at each stage to attain high yields of the two rice varieties. This method enables real-time monitoring with UAVs and adapts well to southwest China. It demonstrates the potential for precise nitrogen management across the ecological types of rice in the region. Future research should focus on advancing multi-source remote sensing technologies for dynamic crop nutrition monitoring to improve the prediction accuracy of the NNI inversion model while implementing multi-site trials across diverse agroecological regions to systematically validate and adapt nitrogen topdressing schemes that ensure precision agriculture practices aligned with sustainable intensification goals.

## Figures and Tables

**Table 1:** Soil physical and chemical properties and planting details of the two experimental sites.

Soil and Crop Information	MZF (2021)	BPS (2022)
Soil pH	5.30	7.70
Organic matter (mg/kg)	23.70	22.61
Total N (g/kg)	1.20	1.25
Olsen-K (mg/kg)	52.20	88.45
Olsen-P (mg/kg)	37.70	4.30
Transplanting date	15 January	15 March
Rice cultivars	Japonica rice (YJ-37)	Indica rice (JY-6135)

MZF: The site for testing situated in Menghai within Yunnan province, BPS: The site for testing situated in Chongqing Beibei.

**Table 2:** Fertilizer application amounts at different test sites (kg·ha^−1^).

Treatment	MZF			BPS		
	N	P_2_O_5_	K_2_O	N	P_2_O_5_	K_2_O
N0	0	120	105	0	120	105
N1	60	120	105	60	120	105
N2	120	120	105	120	120	105
N3	160	120	105	160	120	105
N4	200	120	105	200	120	105

MZF: The site for testing situated in Menghai within Yunnan province, BPS: The site for testing situated in Chongqing Beibei.

**Table 3:** Aircraft parameters.

Parameter	Value
Weight/g	1487
Maximum flight altitude/m	6000
Maximum horizontal flight speed/(kg·h^−1^)	50
Flight time/min	27
Operating ambient temperature/°C	0~40
Hover accuracy/m	Vertical: ±0.1; Horizontal: ±0.3
Image sensor	CMOS
Number of bands	5
Maximum photo resolution/Pixel	1600 × 1300
Working environment temperature/°C	0~40
Multi-spectral band ± range	450 ± 16 nm, 560 ± 16 nm, 650 ± 1 6 nm, 730 ± 16 nm, 840 ± 26 nm

**Table 4:** Vegetation index used for NNI inversion.

Vegetation Index	Formula	References
Normalized differential vegetation index (NDVI)	(ρ NIR − ρ R)/(ρ NIR + ρ R)	[27]
Normalized vegetation index with red edge (NNVI)	(ρ NIR − ρ R)/(ρ NIR + ρ R) × ρ NIR	[28]
Ratio vegetation index (RVI)	ρ NIR/ρ R	[29]
N reflectance index (NRI)	(ρ G − ρ R)/(ρ G + ρ R)	[30]
Ratio vegetation index − 1 (RVI − 1)	ρ NIR/ρ R − 1	[15]
Simple ratio index (SR)	ρ NIR/ρ G	[25]
Normalized differential red marginal vegetation index (NDRE)	(ρ NIR − ρ R)/(ρ NIR + ρ R)	[31]
Normalized color difference index (PSND c)	(ρ NIR − ρ B)/ρ NIR + ρ B)	[15]
Soil regulates vegetation index (SAVI)	1.5 × (ρ NIR − ρ R)/(ρ NIR + ρ R + 0.5)	[32]

ρ NIR. ρ R, ρ G, ρ B represent the reflectance of the near-infrared band, red band, green band and blue wave band of the rice canopy, respectively.

**Table 5:** Basic data of rice field fertilizer application based on NNI.

Variety	Treatment	N Rate (kg·ha^−1^)	TAN (kg·ha^−1^)	APGN (kg)	Yield (kg·ha^−1^)
YJ-37	N0	0	128.90 c	1.61 a	8026.6 e
	N1	60	145.30 bc	1.57 a	9253.3 d
	N2	120	169.40 abc	1.75 a	9680.0 c
	N3	160	178.40 ab	1.74 a	10,280.0 b
	N4	200	208.80 a	1.97 a	10,573.3 a
JY-6135	N0	0	118.56 d	1.93 a	6128.0 d
	N1	60	149.58 c	1.98 a	7553.4 c
	N2	120	169.75 b	2.08 a	8175.8 b
	N3	160	198.01 a	2.11 a	9376.0 a
	N4	200	202.34 a	2.13 a	9505.8 a

Different letters following the values denotes significant differences at 0.05.

**Table 6:** N application rate based on NNI-regulated N application method (kg·ha^−1^).

Variety	Treatment	Basal Fertilizer (kg·ha^−1^)	JT Topdressing (kg·ha^−1^)	HD Topdressing (kg·ha^−1^)	Total N (kg·ha^−1^)
YJ-37	N0	0	0	0	0
	N1	24	74.16 ± 8.51	37.08 ± 8.73	135.24 ± 17.24
	N2	48	78.33 ± 8.51	39.16 ± 8.73	165.49 ± 17.24
	N3	64	82.43 ± 2.41	41.22 ± 4.76	187.65 ± 7.17
	N4	80	74.17 ± 0.00	37.09 ± 2.50	191.26 ± 2.50
JY-6135	N0	0	—	—	0
	N1	24	53.16 ± 29.44	26.58 ± 5.53	103.74 ± 34.97
	N2	48	57.39 ± 29.44	28.69 ± 4.45	134.08 ± 33.89
	N3	64	88.65 ± 0.00	44.33 ± 4.05	196.98 ± 4.05
	N4	80	77.99 ± 0.00	38.99 ± 2.52	196.98 ± 2.52

The values are the mean ± RMSE. JT: The jointing stage, HD: The heading stage.

## Data Availability

The original contributions presented in this study are included in the article. Further inquiries can be directed to the corresponding author.

## Appendix A

The statistical software SPSS 27.0 was used for one-way analysis of variance (ANOVA) with the least significant difference (LSD) test at the 5% level.

**Table A1:** Nitrogen content in rice organs at different test sites (%).

Growth	Treatment	MZF (Cultivar: YJ-37)				BPS (Cultivar: JY-6135)			
		Stem	Leaf	Grain	Plant	Stem	Leaf	Grain	Plant
TR	N0	1.09 ± 0.02 c	2.55 ± 0.09 d	—	2.04 ± 0.06 d	1.47 ± 0.04 c	3.18 ± 0.09 d	—	2.50 ± 0.08 d
	N1	1.26 ± 0.03 b	2.84 ± 0.08 c	—	2.30 ± 0.06 c	1.77 ± 0.07 b	3.50 ± 0.19 c	—	2.83 ± 0.1 c
	N2	1.40 ± 0.06 b	3.12 ± 0.05 b	—	2.51 ± 0.05 b	1.83 ± 0.06 b	3.74 ± 0.15 b	—	3.08 ± 0.19 b
	N3	1.66 ± 0.06 a	3.43 ± 0.15 a	—	2.78 ± 0.12 a	1.97 ± 0.13 a	4.04 ± 0.15 a	—	3.23 ± 0.18 ab
	N4	1.74 ± 0.24 a	3.46 ± 0.30 a	—	2.82 ± 0.23 a	2.01 ± 0.12 a	4.09 ± 0.05 a	—	3.32 ± 0.10 a
JT	N0	0.88 ± 0.01 d	2.36 ± 0.02 c	—	1.43 ± 0.04 d	0.92 ± 0.04 e	2.20 ± 0.07 d	—	1.52 ± 0.05 d
	N1	0.93 ± 0.01 c	2.44 ± 0.04 c	—	1.55 ± 0.04 c	1.05 ± 0.02 d	2.46 ± 0.02 c	—	1.74 ± 0.02 c
	N2	1.08 ± 0.04 b	2.79 ± 0.02 b	—	1.68 ± 0.04 b	1.23 ± 0.09 c	3.01 ± 0.09 b	—	2.09 ± 0.03 b
	N3	1.16 ± 0.04 a	2.94 ± 0.11 a	—	1.81 ± 0.07 a	1.49 ± 0.03 b	3.25 ± 0.13 a	—	2.35 ± 0.08 a
	N4	1.14 ± 0.02 a	2.89 ± 0.04 a	—	1.77 ± 0.04 a	1.39 ± 0.01 a	3.34 ± 0.16 a	—	2.34 ± 0.12 a
HD	N0	0.76 ± 0.05 c	2.26 ± 0.08 c	1.00 ± 0.05 d	1.31 ± 0.04 c	0.46 ± 0.02 d	1.88 ± 0.03 d	0.94 ± 0.07 c	0.92 ± 0.03 d
	N1	0.85 ± 0.01 b	2.55 ± 0.02 b	1.13 ± 0.02 c	1.42 ± 0.02 b	0.58 ± 0.03 c	2.19 ± 0.15 c	1.03 ± 0.02 b	1.11 ± 0.05 c
	N2	0.89 ± 0.02 b	2.61 ± 0.03 b	1.25 ± 0.05 b	1.46 ± 0.02 b	0.72 ± 0.01 b	2.58 ± 0.06 b	1.06 ± 0.06 ab	1.33 ± 0.02 b
	N3	1.05 ± 0.09 a	2.95 ± 0.13 a	1.46 ± 0.06 a	1.73 ± 0.08 a	0.84 ± 0.03 a	2.83 ± 0.07 a	1.11 ± 0.07 a	1.48 ± 0.05 a
	N4	1.02 ± 0.04 a	2.98 ± 0.12 a	1.49 ± 0.05 a	1.70 ± 0.08 a	0.84 ± 0.03 a	2.61 ± 0.12 b	1.09 ± 0.04 bc	1.48 ± 0.05 a
FL	N0	0.77 ± 0.02 c	1.65 ± 0.08 c	1.14 ± 0.02 b	1.22 ± 0.02 b	0.33 ± 0.01 c	1.45 ± 0.03 c	0.79 ± 0.02 c	0.74 ± 0.01 c
	N1	0.80 ± 0.05 c	1.85 ± 0.19 b	1.19 ± 0.04 b	1.29 ± 0.09 b	0.39 ± 0.01 c	1.66 ± 0.10 b	0.84 ± 0.04 b	0.82 ± 0.01 b
	N2	0.95 ± 0.04 b	2.30 ± 0.09 a	1.35 ± 0.04 a	1.52 ± 0.06 a	0.46 ± 0.05 b	1.95 ± 0.08 a	0.93 ± 0.05 a	0.95 ± 0.02 a
	N3	1.04 ± 0.05 a	2.33 ± 0.03 a	1.40 ± 0.08 a	1.54 ± 0.04 a	0.53 ± 0.07 a	2.04 ± 0.08 a	0.88 ± 0.02 b	0.97 ± 0.03 a
	N4	0.93 ± 0.08 b	2.30 ± 0.05 a	1.34 ± 0.06 a	1.48 ± 0.05 a	0.53 ± 0.02 b	1.95 ± 0.05 a	0.88 ± 0.04 b	0.96 ± 0.01 a

Different letters following the values denotes significant differences at 0.05.

**Table A2:** Critical aboveground nitrogen content of different rice varieties (%).

Growth	YJ-37	JY-6135
	Critical Aboveground N Concentration	Critical Aboveground N Concentration
TR	2.68	3.87
JT	1.72	2.01
HD	1.64	1.20
FL	1.38	0.94
